# Best practice for analysis of shared clinical trial data

**DOI:** 10.1186/s12874-016-0170-y

**Published:** 2016-07-08

**Authors:** Sally Hollis, Christine Fletcher, Frances Lynn, Hans-Joerg Urban, Janice Branson, Hans-Ulrich Burger, Catrin Tudur Smith, Matthew R. Sydes, Christoph Gerlinger

**Affiliations:** AstraZeneca, Alderley Park, Cheshire, SK10 4TG UK; Centre for Biostatistics, Institute of Population Health, University of Manchester, Manchester Academic Health Science Centre, Oxford Road, Manchester, M13 9PL UK; Amgen Ltd., 240 Cambridge Science Park, Cambridge, CB4 0WD UK; BioMarin, 10 Bloomsbury Way, London, WC1A 2SL UK; Hoffman-La Roche, Grenzacherstrasse 124, 4070 Basel, Switzerland; Novartis Pharma AG, Basel, Switzerland; MRC North West Hub for Trials Methodology Research, University of Liverpool, Liverpool, UK; MRC Clinical Trials Unit at UCL, Institute of Clinical Trials & Methodology, Aviation House, 125 Kingsway, London, WC2B 6NH UK; MRC London Hub for Trials Methodology Research, Aviation House, 125 Kingsway, London, WC2B 6NH UK; Research and Development Statistics, Bayer Pharma AG, 13353 Berlin, Germany; Gynecology, Obstetrics and Reproductive Medicine, University Medical School of Saarland, 66421 Homburg/Saar, Germany

## Abstract

**Background:**

Greater transparency, including sharing of patient-level data for further research, is an increasingly important topic for organisations who sponsor, fund and conduct clinical trials. This is a major paradigm shift with the aim of maximising the value of patient-level data from clinical trials for the benefit of future patients and society. We consider the analysis of shared clinical trial data in three broad categories: (1) reanalysis - further investigation of the efficacy and safety of the randomized intervention, (2) meta-analysis, and (3) supplemental analysis for a research question that is not directly assessing the randomized intervention.

**Discussion:**

In order to support appropriate interpretation and limit the risk of misleading findings, analysis of shared clinical trial data should have a pre-specified analysis plan. However, it is not generally possible to limit bias and control multiplicity to the extent that is possible in the original trial design, conduct and analysis, and this should be acknowledged and taken into account when interpreting results. We highlight a number of areas where specific considerations arise in planning, conducting, interpreting and reporting analyses of shared clinical trial data. A key issue is that that these analyses essentially share many of the limitations of any *post hoc* analyses beyond the original specified analyses. The use of individual patient data in meta-analysis can provide increased precision and reduce bias. Supplemental analyses are subject to many of the same issues that arise in broader epidemiological analyses. Specific discussion topics are addressed within each of these areas.

**Summary:**

Increased provision of patient-level data from industry and academic-led clinical trials for secondary research can benefit future patients and society. Responsible data sharing, including transparency of the research objectives, analysis plans and of the results will support appropriate interpretation and help to address the risk of misleading results and avoid unfounded health scares.

## Background

Greater transparency, including sharing of patient-level data for further research, is an increasingly important topic for the pharmaceutical industry and other organisations (government agencies, academia, charities etc.) who sponsor, fund and conduct clinical trials. Drivers of these changes have come from several sources – for example, the scientific community/academia [1, 2], regulators [3–5], and the pharmaceutical industry [6]. This paradigm shift aims to maximise the value of patient-level data from clinical trials for the benefit of future patients and society, by sharing clinical trial data for secondary research. However, the risk of publication of misleading results and unfounded health scares has also been recognized by those advocating increased access [3]; responsible data sharing, including transparency of the research requests, objectives, analysis plans and results will support appropriate interpretation and help to mitigate this risk. This article is one of five related articles in this journal, resulting from a working group formed by EFSPI (European Federation of Statisticians in the Pharmaceutical Industry) and PSI (Statisticians in the Pharmaceutical Industry) to examine various aspects of transparency of patient-level data from clinical trials. The focus here is on analysis of shared data from trials sponsored by pharmaceutical companies, but the principles discussed also apply more broadly.

We will consider analysis of shared clinical data within three broad categories:

i. Reanalysis: further investigation of the efficacy and safety of the randomized intervention using individual patient-level data from a clinical trial, e.g.
  Using a new measure of benefit or risk that can be derived from the available data
  Exploring the impact of analysis assumptions made, such as the handling of missing data
  Verification of the results in the original study report or publication
ii. Meta-analysis: further investigation of the efficacy and safety of one or more randomized interventions using individual patient-level data from several clinical trials, e.g.
  meta-analysis to learn more about an intervention by pooling several trials including the same comparison
  network meta-analysis to learn more about the relative effect of various interventions by making indirect comparisons across several trials with different comparators
iii. Supplemental analysis: use of individual patient-level data from a clinical trial for a research question that is not directly assessing the randomized intervention, e.g.
  exploring prognostic factors and characterising disease evolution over time
  evaluating new statistical methods
  understanding relationships between endpoints
  gaining information to inform the design of a future study

This paper will lay out some key considerations relevant to planning, conducting, interpreting and reporting analyses of shared clinical trial data, and then address issues specifically relevant to each of the categories above.

Existing methodological guidelines for analysis of randomized trials, meta-analysis of randomized trials, or analysis of epidemiological data can be used for general guidance for best practice on what analysis features should be pre-specified and how the results should be reported. This debate paper aims to highlight specific areas where additional considerations may arise.

## Discussion

### General considerations for the analysis of shared clinical trial data

The original analysis of a clinical trial data provides protection against bias and misinterpretation by pre-specification of the objectives and the analysis methods. In order to support appropriate interpretation and limit the risk of misleading findings, analyses of shared clinical trial data should also have a pre-specified analysis plan. However, it is not generally possible to limit bias and control multiplicity to the extent that is possible in the original trial design, conduct and analysis, and this should be acknowledged and taken into account when interpreting the results of analyses of shared clinical trial data. The nature of the potential biases and sources of multiplicity will differ between the scenarios considered, and so this topic is addressed in more detail in the relevant sections below.

#### Planning the research

The data requestor should establish the data holder’s expectations of the data sharing process, including the process of handling queries at the planning and conduct stages, and any expectations for the data requestor to inform the data holder of their findings and publication plans. Some data holders may offer a specific point of contact who can provide advice, whilst others may have established systems for dealing with any queries (e.g. ClinicalStudyDataRequest.com). The data holder will generally be able to provide documentation which will help the data requestor understand the trial design, conduct and analysis methods (including the protocol, statistical analysis plan and reports), and the data structures (such as annotated case report forms and database specifications). Some data holders may be willing to share some or all of this documentation with a data requestor to support their planning, and some will provide full documentation only once a research proposal has been agreed. Requestors should ensure that they set aside time for navigating the access process and be aware that they will be required to agree to a legal data use agreement. Further guidance on important points for data requestors to consider and guidance on the most efficient way to obtain the information needed is covered in a companion article in this collection [7].

The data requestor should define and document the question of interest, hypotheses, and the planned statistical analyses before accessing the data. While the design of the study and the raw data collected are fixed, there are many aspects of the planed analysis which can be pre-specified. An analysis plan should generally include details of:

- The main objectives
- The populations and variables to be analysed, including details of any subjects and data which will be excluded
- Statistical tests or models to be used, including covariates where applicable
- Any data transformations to be used, and how any missing data or outliers will be handled
- Any planned adjustment of significance and confidence levels
- Any planned sensitivity analyses to explore the robustness of the results
- Any planned investigation of subgroups

Analytical methods that are inherently exploratory in nature such as data mining can also be of value in this context, provided that the intended purpose and scope are clearly defined.

At the planning stage, dialogue with the data holder is recommended to ensure that the data being requested can support the intended analyses. For example a data requestor may need to know whether planned covariates will be available in the datasets supplied, i.e. that the information has been collected at the required level of detail, and whether it will be subject to any redaction due to data privacy provisions. Many data holders will routinely apply a number of de-identification techniques prior to sharing individual patient level data [8]. These commonly include masking absolute dates from datasets and/or retaining information about the relative timing of events only, and censoring ages, for example above 89 years. In some cases, data privacy rules in different regions may lead to selected patients being removed entirely from the datasets. The impact of this should be considered and discussed with the data holder where necessary. For example, if the question of interest involves the seasonal pattern of events such as asthma exacerbations, then the data requestor should discuss with the data holder whether data could be provided that retain sufficient information for this to be further investigated.

Ideally the protocol and analysis plan for research on shared clinical trial data should be publically documented. This helps to prevent unplanned duplication of research, and allows the level of pre-specification of analyses to be subsequently verified. In some cases, the data holder may make provisions for this as part of the data request process, or there may be opportunities such as the PROSPERO database [9] or Cochrane library for meta-analysis protocols, or within journals which are increasingly providing opportunities for the publication of research protocols.

#### Conducting the analyses

Once a data requestor obtains access to the data, a key step prior to performing analyses is to understand the data including the data structure, formats, coding used, the meaning of each variable in the data set, and establishing which variables reflect ‘raw’ data from the case report forms, medical notes or direct results of trial assessments, and which variables reflect ‘derived’ variables where algorithms have been applied. Dialogue with the data holder may be needed during conduct of the analyses, particularly for older trials where the complete documentation may not be available, or may be lacking in detail.

It is best practice for the data recipient to replicate the primary analysis of the primary endpoint of the study where this is possible. This allows the data recipient to increase their confidence in understanding the structure and format of the data set before proceeding to conduct their planned analyses. Where an exact replication of the original results will not be possible due to data privacy provisions, the data recipient may wish to request that the data holder provides the results obtained by them when the planned primary analysis is run on the shared data, in order that this check can still be applied.

The data recipient should also check that the proposed analyses can be carried out. If necessary, depending on the structure of the data received or on initial explorations of the data, amendments could be made to the analysis plan. There should be clear documentation of any amendments to the methods and analyses, including details of the scientific rationale and the timing of changes relative to access to the datasets. If a data recipient wishes to make a significant expansion to the scope of the research, such as the consideration of additional derived endpoints, the data holder should be contacted to submit an expanded request.

#### Interpretation

The data recipient should consider any potential sources of bias in the analyses due to features of the design and conduct of the trial, and take these into account when interpreting the results of further analyses, such as combining blinded trials with open trials in a meta-analysis or using hazard ratios to combine trials where one or more trials have pronounced non-proportional hazards and have used a different summary measure.

As with the original analyses, clinical relevance needs to be considered as well as statistical significance; sensitivity analyses may also prove useful to help demonstrate the robustness of the results.

If a data recipient identifies a potential new safety signal then it is important that they immediately inform any parties responsible for the intervention. This would generally include the sponsor of the trial. For licenced medicines, the market authorization holder will have processes in place to ensure appropriate consideration and handling of potentially important new safety findings, including communication to the relevant competent authorities (EMA, FDA, etc.) where necessary.

#### Reporting

For full transparency and to avoid publication bias, data requestors conducting analyses on shared clinical trial data must make all attempts to publish all results of the pre-specified analyses in a peer reviewed journal, regardless of the findings. In line with good trial reporting practice [10], all publications should state that the results were generated after the completion of the original clinical trial analysis and reporting, including references to publications containing the results from the original analyses. Any new objectives or analyses that were added after access to the data was obtained should be clearly identified as such and interpreted appropriately in light of this.

If the trial is registered on suitable clinical trials registries, the corresponding identifier should be included in any publications (e.g. ClinicalTrials.gov and “NCT Number”). There is an automatic identification process whereby publications indexed in MEDLINE are linked to the trial record [11].

Some analyses of shared clinical trial data may not be of great interest to journals, e.g. further analyses of the efficacy or safety of any intervention that provide little new clinically relevant information, or a simple analysis to support sample sizing. Either the data recipient’s or the data requestor’s institution may have mechanisms which could be used for posting such information in the public domain as reports or “white papers”.

### Specific considerations for reanalysis

#### Overview of potential biases and sources of multiplicity

ICH (International Council for Harmonisation of Technical Requirements for Pharmaceuticals for Human Use) E9 ‘Statistical Principles for Clinical Trials’ is a key guidance for statistical methodology applied to clinical trials for marketing applications demonstrating the safety and efficacy of medicinal products [12]. Throughout the ICH E9 guidance there is emphasis on the use of pre-specification to protect against multiplicity of methods and interpretation and of blind review (checking of data prior to the breaking of the blind) in order that unbiased decisions about the analysis methods can be made. For confirmatory trials of medicines, key statistical methods will be defined in the protocol prior to initiation of the trial, and a statistical analysis plan will be written prior to un-blinding of the data. The analysis plan will provide details of the analysis populations, the derivation of variables, and the statistical methods, and document any changes from the analyses planned in the protocol. The level of detail contained in a confirmatory trial analysis plan has increased over recent decades, and it is now common for these plans to be from tens to hundreds of pages long. Multiplicity is addressed through the hierarchy of primary, secondary and exploratory objectives. Formal control of the type 1 error may be specified across multiple primary and secondary variables, multiple comparisons of treatments, and repeated evaluations over time. This framework of pre-planned analyses supports appropriate interpretation of the results. There will also be an evaluation of the robustness of the results and primary conclusions of the trial, examining the sensitivity of the overall conclusions to various limitations of the data, assumptions made, and choice of analysis methods. Key sensitivity analyses will be pre-specified in the analysis plan, but the need for further exploratory analyses may become apparent on further examination of the data. Even in the presence of a thorough analysis plan, unexpected findings can lead to a need for post-hoc analyses. Regardless of what motivates such post-hoc analyses, they are likely to produce biased results, and introduce multiplicity issues that cannot easily be controlled. Publication bias is also more likely to apply to post-hoc analyses.

Analyses of shared clinical trial data are essentially *post hoc* analyses, and therefore of exploratory, rather than confirmatory, value. While new medical insights can be generated from *post hoc* analyses, it will be especially important to consider broader factors when interpreting the results and discussing whether causation might be concluded from an observed association [13].

Some data recipients may have a direct interest in refuting the interpretation of the original analyses. This could be a source of bias, but would still be best dealt with through the usual scientific process of evaluation of evidence which would include the application of the recommendations for planning, conduct, interpretation and reporting described in this debate article. However, even when data recipients do not have any such direct agenda, there is still increased potential for bias in analyses of shared clinical trial data compared to the original analyses conducted. The original results will generally have been published prior to data being shared and so the data recipient is likely to be aware of the main findings and even some information such as the amount and nature of missing data in each randomized group. As such, further analyses should generally be considered subject to potential bias in the same way that post hoc analyses performed by the original researchers would be. The interpretation of reanalysis can be strengthened by explicitly stating in the research proposal and analysis plan the basis for any differences from the original analysis methods, and the extent to which information relating to the likely impact is already known.

#### Subgroup analyses

The issues in interpretation of subgroup analyses beyond those planned in the original study analysis apply to both post hoc analyses by the original researchers, and to analyses of shared clinical trial data. There is interest from regulatory authorities, patients, medical practitioners and Health Technology Assessors in whether the effects of a medicine are consistent across a range of patient subsets. However, the dangers of over-interpreting findings from exploratory subgroups including the risk of false positives and the low power of interaction tests are well documented in the literature [14, 15]. ICH E9 [12] notes that in most case subgroups investigations are exploratory in nature, and should be interpreted cautiously. This also applies to an investigation of a subgroup in an analysis of shared clinical trial data, and the potential for significant bias needs to be made clear when interpreting and reporting the results [16, 17]. The biological plausibility of a subgroup effect should be considered [13]. Subgroup analyses should not be fishing expeditions to find a group where the treatment is effective or ineffective.

#### Applying alternative analytical approaches

Since the clinical trial data were collected specifically to address the objectives of the original protocol, there may be limitations in the data being able to fully address additional clinical questions of interest. This may be due broadly to the way participants were selected or specifically due to the way in which data were recorded or coded for analysis. For example, a new analysis may be planned where the original outcome definition is modified to only include events which led to a specific medical intervention. The clinical relevance of this new definition should be justified, and the limitations of using medical intervention data which were not collected for this purpose should be considered. Attenuation of the estimated effect of the intervention could occur if there is any misclassification of the medical intervention. Also, the precision could be reduced, for example if a new outcome leads to fewer events.

Attention should be paid to how missing data will be handled, clearly distinguishing whether the missing data are already inherent in the original study data, and thus have been treated or reported in the original Clinical Study Report (CSR), or the missing data are due to the data sharing and data transfer process, such as the categorization of age. In the first case missing data could be treated according to predefined imputation rules either as given in the original protocol or Statistical Analysis Plan (SAP), or according to newly defined rules in the data recipients’ analysis plan. In either case, sensitivity analyses are recommended to evaluate the possible impact of the missing data and the methods used to handle missing data on the outcome in comparison to the original results. Sensitivity analyses have to be planned and conducted carefully – (e.g. Thabane et al [18]). Methodology has evolved in recent years, for example Morris et al [19] and Mallinckrodt et al [20] give good examples of dealing with missing data assumptions within a sensitivity analyses framework.

Additionally, if the original analysis was carried out using a statistical model with covariates, it may be interesting to explore the effect of a different set of covariates or of fitting no covariates. However, when changing the covariates in a model, consideration should be given to the biological plausibility of the variables.

Where the data recipient is applying newer analytical approaches to shared clinical trial data, resulting publications should compare and contrast back to the results obtained from the original clinical trial(s). Possible reasons for differences in results should be described and differences in assumptions used by the different methods clarified. An important aspect to consider is the resolution of conflicting results between the original analysis and any new analyses. During drug licencing, differing analyses are taken into consideration by the regulatory authorities in light of which analyses were primary, were pre-specified as secondary or sensitivity analyses, and which were done post-hoc. The final interpretation of benefit-risk is then taken by the drug licensing authority. If a new analysis potentially alters the benefit-risk profile of a drug, perhaps in a newly-investigated subgroup, it is imperative that the methods, assumptions, and possible reasons for important discrepancies are clearly described. The data holder should be informed of the results, and evaluation of the implications by a licensing authority might sometimes be required.

#### Research aiming to replicate and verify the original results

To date, reanalysis of shared clinical trial data with the primary purpose of replicating and verifying the results of the primary analysis has been uncommon, though some examples do exist. Sydes et al [21] report the experience of the MRC clinical trials unit; of the 103 formal data sharing activities logged in 2012, 2013 and 2014, none aimed to replicate the original analyses. Strom et al [22] report the experience of an independent review panel overseeing requests for access to de-identified patient-level data from clinical trials sponsored by GlaxoSmithKline. In the first year, 23 requests had progressed to a signed data-sharing agreement, with only one of these explicitly identifying concerns about the way in which a study was analysed and reported and aiming to reanalyse the data from the study and report all findings objectively. The results of the reanalysis have subsequently been published [23].

As noted above, an exact replication of the original results might not always be possible, for example due to data privacy provisions. This is particularly problematic when the primary purpose of the reanalysis is to replicate and verify the original results. If there is likely to be any impact of data privacy provisions on the analyses that are to be replicated, the data recipient and data holder should discuss during the request process how this issue will be handled.

When a reanalysis of study data is planned which aims purely to replicate the results in the original study report, it is important to ensure that the analysis population, the outcome measures and the statistical methodology are all consistent with the original plans. A comparison should be carried out between any newly derived and the original derived variables and any discrepancies should be accounted for, where possible.

It is recommended that if differences are observed that impact the interpretation of the results, then data recipients discuss the reanalysis with the authors of the original analyses prior to publication of their findings. This can help to understand the reason behind any differences, and hence avoid causing any unnecessary concern which could arise.

### Specific considerations for meta-analysis

With increasing data transparency, data recipients are increasingly able to conduct meta-analysis (MA) and network meta-analysis (NMA) using individual patient data (IPD) rather than using aggregate data obtained from publications. The use of IPD in MA and NMA may increase precision of the effects being estimated, and examination of subgroups across multiple studies increases power and allows the consistency of effects to be assessed. In particular, the use of IPD limits possible ecological bias [24–26]. For an NMA, the use of patient-level covariates should also allow a better evaluation of the heterogeneity and inconsistency in the network [27].

There are a number of general guidance documents which describe good practices for planning, conducting reporting and interpreting MA [28, 29] and NMA [30–32]. In MA and NMA there are key assumptions including exchangeability, homogeneity and similarity of trials being pooled, and for NMA the consistency of direct and indirect evidence. Research proposals for MA and NMA need to describe the clinical evidence forming the basis for the analysis, possible biases and limitations of the available evidence, the statistical methods to be used, and a range of sensitivity analyses that enable the robustness of the results to be assessed relative to the various assumptions being made. Bender et al [33] describe sources of multiplicity which commonly arise in meta-analyses, including multiple outcomes, multiple interventions, multiple time points, sub-group analyses and accumulating data.

However there are a number of additional considerations when conducting MA or NMA using IPD [29, 32, 34, 35]. For example, there may be potential bias if IPD cannot be made available for all studies [36]; inconsistencies in IPD may arise when studies use different variable definitions, or adopt different randomization and follow-up procedures; statistical method used (e.g. one-stage versus two-stage models). In some cases, a combination of aggregate (summary) data and IPD data may be available for the MA or NMA. There are different approaches available for combining aggregate data and IPD in an MA and NMA, with research ongoing to establish potential biases and limitations in the analytical approaches [27, 37].

Some data holders require use of a locked analysis system, which allows upload but not export of data. This would not place any limitations on a two-stage analysis approach in which the IPD are analysed consistently but separately to produce trial-specific estimates of effects of interest which are then combined in a weighted average. However, this could pose challenges for a one-stage approach where all trials are analysed simultaneously within a single statistical model. We note that some sponsors are participating in multi-sponsor environments (e.g. the Clinical Data Study Request site [38] and the YODA project [39]) that allow for analyses across sponsors. Where this is not the case, dialogue with the various data holders is recommended to seek a collaborative solution that is acceptable to all parties. Additionally, a central repository has been proposed for IPD that have already been collected by collaborative groups to ensure that these datasets are safeguarded and made available for use by others [40].

### Specific considerations for supplemental analysis

Examples of research objectives in this category include:

- exploring prognostic factors and characterising disease evolution over time
- evaluating new statistical methods
- understanding relationships between endpoints
- gaining information to inform the design of a future study

When making any comparison between non-randomized groups of subjects, the most critical issue to consider will be the control of bias and confounding. Existing methodological guidelines for epidemiological analyses can be used for guidance on available analysis methods, what features should be pre-specified, and how the results should be reported [41]. There are a number of areas where further guidance would be valuable, and we welcome the strengthening analytical thinking for observational studies (STRATOS) initiative, a large collaboration of experts in many different areas of biostatistical research with the objective to provide accessible and accurate guidance in the design and analysis of observational studies [42].

When completely new research questions are addressed, there may be opportunities for confirmatory analysis with formal multiplicity control as a technique to control the risk of false positive findings. Even when more exploratory analyses are planned, pre-specification of the objectives and methods is still valuable to aid interpretation.

When clinical trial data are used for a research question that is not directly assessing the randomized intervention, the systematic effect that is introduced by the randomisation may need to be considered. The intervention assignment may generate spurious relationships, or mask relationships within the data. It will generally be important to allow for the intervention assignment by performing analysis within each arm of the trial, or restricting the analysis to a single arm. Alternatively the intervention assignment could be included as a covariate along with relevant interaction terms in a statistical model, though this complexity will still need to be addressed in the interpretation.

## Summary

Increased provision of patient-level data from industry and academic-led clinical trials for secondary research can benefit future patients and society. The risk of publication of misleading results and the potential for unfounded health scares has been highlighted [3]; responsible data sharing, including transparency of the research objectives, analysis plans and the results will support appropriate interpretation and help to address this risk.

In order to support appropriate interpretation and limit the risk of misleading findings, analysis of shared clinical trial data should have a pre-specified analysis plan. However, it is not generally possible to limit bias and control multiplicity to the extent that is possible in the original trial design, conduct and analysis, and this should be acknowledged and taken into account when interpreting results. Following the best practices highlighted in this paper can increase the validity of these analyses; ensuring there are appropriate planned statistical analyses, that the existing data can support these, that results are appropriately presented and interpreted, and appropriately disseminated to the research community. A key issue is that all analyses of shared clinical trial data essentially share many of the limitations of any *post hoc* analyses beyond the original specified analyses. The use of individual patient data in meta-analysis can provide increased precision and reduce bias. Supplemental analyses are subject to many of the same issues that arise in broader epidemiological analyses.

There are many remaining challenges in achieving responsible data sharing. The Institute of Medicine conclude their comprehensive review of this area by recommending the convening of a multi-stakeholder body to address the key infrastructure, technological, sustainability, and workforce challenges associated with the sharing of clinical trial data [43].

## Abbreviations

CSR: Clinical Study Report, EFSPI: European Federation of Statisticians in the Pharmaceutical Industry, EMA: European Medicines Agency, FDA: Food and Drug Administration, ICH: International Council for Harmonisation of Technical Requirements for Pharmaceuticals for Human Use, IPD: individual patient data; MA: meta-analysis; NMA: network meta-analysis, PSI: Statisticians in the Pharmaceutical Industry, SAP: Statistical Analysis Plan, STRATOS: strengthening analytical thinking for observational studies.
